# Uncovering the Profile of Somatic mtDNA Mutations in Chinese Colorectal Cancer Patients

**DOI:** 10.1371/journal.pone.0021613

**Published:** 2011-06-28

**Authors:** Cheng-Ye Wang, Hui Li, Xiao-Dan Hao, Jia Liu, Jia-Xin Wang, Wen-Zhi Wang, Qing-Peng Kong, Ya-Ping Zhang

**Affiliations:** 1 State Key Laboratory of Genetic Resources and Evolution, Kunming Institute of Zoology, Chinese Academy of Sciences, Kunming, China; 2 Laboratory for Conservation and Utilization of Bio-resource, Yunnan University, Kunming, China; 3 KIZ/CUHK Joint Laboratory of Bioresources and Molecular Research in Common Diseases, Kunming, China; 4 Graduate School of the Chinese Academy of Sciences, Beijing, China; University of Medicine and Dentistry of New Jersey, United States of America

## Abstract

In the past decade, a high incidence of somatic mitochondrial DNA (mtDNA) mutations has been observed, mostly based on a fraction of the molecule, in various cancerous tissues; nevertheless, some of them were queried due to problems in data quality. Obviously, without a comprehensive understanding of mtDNA mutational profile in the cancerous tissue of a specific patient, it is unlikely to disclose the genuine relationship between somatic mtDNA mutations and tumorigenesis. To achieve this objective, the most straightforward way is to directly compare the whole mtDNA genome variation among three tissues (namely, cancerous tissue, para-cancerous tissue, and distant normal tissue) from the same patient. Considering the fact that most of the previous studies on the role of mtDNA in colorectal tumor focused merely on the D-loop or partial segment of the molecule, in the current study we have collected three tissues (cancerous, para-cancerous and normal tissues) respectively recruited from 20 patients with colorectal tumor and completely sequenced the mitochondrial genome of each tissue. Our results reveal a relatively lower incidence of somatic mutations in these patients; intriguingly, all somatic mutations are in heteroplasmic status. Surprisingly, the observed somatic mutations are not restricted to cancer tissues, for the para-cancer tissues and distant normal tissues also harbor somatic mtDNA mutations with a lower frequency than cancerous tissues but higher than that observed in the general population. Our results suggest that somatic mtDNA mutations in cancerous tissues could not be simply explained as a consequence of tumorigenesis; meanwhile, the somatic mtDNA mutations in normal tissues might reflect an altered physiological environment in cancer patients.

## Introduction

In the past decade, the role of mitochondrial DNA (mtDNA) mutation in tumorigenesis has received much attention. A high incidence of somatic mtDNA mutations, mostly in homoplastic, has been identified in nearly all types of cancerous tissues [1], [2]. Nonetheless, the relationship of somatic mtDNA mutations and tumorigenesis has been hotly debated. Some studies suggested that the mtDNA somatic mutations likely result from the elevated reactive oxygen species (ROS) in tumor cell [3], [4], [5], because most of these mutations are T to C and G to A base transitions [1], [2], similar to the mutation pattern of oxidative decay on DNA [6], [7]. It was also suggested that some changes in mtDNA could disable the function of mitochondrial respiratory chain and thus contribute to tumor procession [8], [9], [10], [11]. For example, a recent study suggested that mtDNA mutations which produce the deficiency in respiratory complex I activity could contribute to tumor progression by enhancing the metastatic potential of tumor cells [12]. In contrast, some studies suggested that the somatic mutation in tumor tissue could simply arise by chance in tumor progenitor cells without any physiological advantage or tumorigenic requirement [13], [14]. Indeed, Vega and colleagues found that a lot of mtDNA mutations detected in tumor were in fact the common polymorphisms or mutational hotspots prevailing in human general populations, leading the authors to propose that these somatic mutations are more likely to be neutral [15]. Similarly, the functional roles of some of the identified somatic mtDNA mutations were queried largely due to the problems in data quality [16], [17], [18]. Obviously, further evidence is needed to elucidate the exact role that somatic mtDNA mutations have play in tumorigenesis.

In the present study, we performed the whole mitochondrial genome screening for somatic mutation in paired cancerous and non-cancerous tissues from 20 patients with colorectal cancer. By using the same quality control measures as adopted in our previous studies in complete mtDNA sequencing [19], [20], [21], [22], we want to get a deeper insight into the profile of the mtDNA somatic mutations in colorectal cancer. The direct comparison of the entire mtDNA sequences of the primary cancerous, matched para-cancerous normal and distant normal tissues from the same patient, accompanied with the stringent data-quality control, is adopted to disclose a detailed profile of mtDNA somatic mutations in both normal and tumor tissues in the same patient. Our results revealed that the observed somatic mutations in our samples are much less frequent than the previous studies. Surprisingly, all the observed somatic mutations are in heteroplasmic state, an observation quite different from the previous studies.

## Result

**Figure 1:**
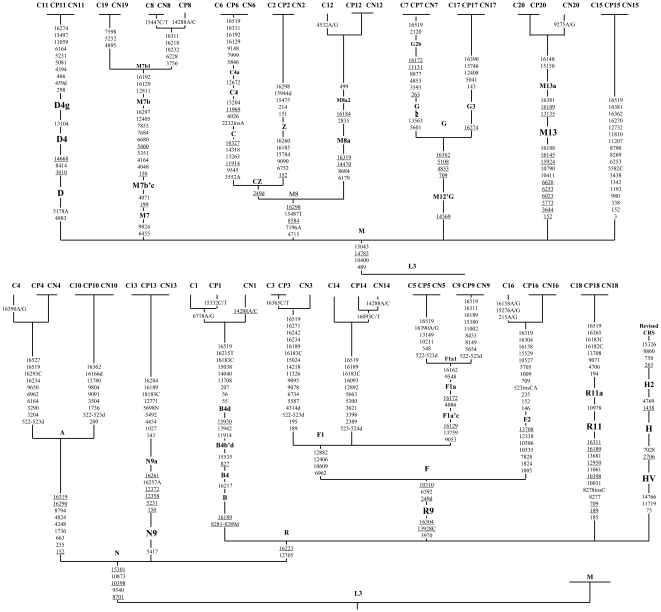
Phylogenetic tree of 59 complete mtDNA sequences from colorectal normal and malignant tissues. Haplogroup names are inserted along the branches determining the locations of the corresponding ancestral haplotypes. Variants (reconstructed most parsimoniously) on uninterrupted branches are listed arbitrarily in ascending order. Suffixes “A, C and T” indicate transversions, “ins” indicates a nucleotide insertion; “C/T, A/C, and A/G” suggest heteroplasmic mutations, “d” indicates a deletion. Recurrent variants are underlined. Length variation of C-tract around positions 310 and 16189 is not indicated in the figure. Suffixes “C, CP, and CN” in the sample names indicate “cancerous tissue, para-cancerous tissue and normal tissue” respectively.

A total of 59 complete mitochondrial genome sequences of three paired tissues from 20 colorectal cancer patients were obtained in this study, and all the sequences have been deposited into GenBank (http://www.ncbi.nlm.nih.gov/Genbank/) with accession numbers GU392048–GU392106. All the mtDNA variable sites of each tissue have been displayed on a schematic tree (Figure 1), which could provide a direct comparison of the entire mtDNA genome variation among three tissues from the same patient and thus allow pinpointing the somatic mutation(s) in a patient conveniently. As demonstrated in Figure 1, six of 20 patients carried mtDNA somatic mutations in their cancerous tissues, occupying a much lesser proportion (6/20; 30%) than many previous reports. For example, Tan et al. found 27 somatic mutations in 14/19 (74%) breast patients [23]; Zhu et al. found 45 somatic mutations at 35 nucleotide position in 14/15 (93%) breast cancer patients [24]; in Polyak et al. (1998), 7/10 (70%) colorectal tumor cases were demonstrated to carry one to three mutations.

In total, there are 18 somatic mutations were detected in the 59 tissue samples under study (Figure 1 and Table S1), and all the somatic mutations occur merely on the terminal branches of phylogenic tree, and no haplotype-shift was observed. With the exception of the RNA-coding genes (including sRNA and tRNA), these somatic mutation sites are dispersed across the entire mtDNA genome, which seems quite different from the mutation spectrum observed in patients with mitochondrial diseases [25], [26]. Specifically, seven mutations locate in the D-loop region, whereas the remaining 11 locate in protein-coding regions. Among the 11 coding-region somatic mutations, only two (i.e., G4532A and A9275G) are synonymous transitions which occur on two unstable sites; whereas the rest nine mutations all are non-synonymous substitutions that locate at positions 6718, 14288, 15332, 15447, and 15276, most of which are evolutionarily highly conserved after compared with the mitochondrial genomes from 13 different vertebrate species (Table S1). By further evaluating the conservation of these five mutational sites among a total of published 3,635 complete mitochondrial genome sequences from general human populations, our results revealed that all of the five sites are highly conserved among the general human populations as well (Table S1). Surprisingly, in spite of the high conservation of site 6718, heteroplasmic variation 6718A/G presents in both the cancer and the para-cancer normal tissues of Patient 1, raising the possibility that the para-cancer normal tissue has likely been invaded by cancer cells. Indeed, a similar pattern is observed in Patient 3, in which its cancer and para-cancer tissues share the same heteroplasmic variation 16365C/T. This observation, again, emphasizes the particular necessity of the inclusion of the distant normal tissue for analysis.

Intriguingly, as shown in Figure 1, the pinpointed mtDNA somatic mutations are not restricted merely to tumor tissues, for we did have observed some somatic mutations in the normal tissues of Patients 1, 3, 8, 14, and 20. Specifically, transition 14288 seems to be a recurrent mutation site because it appears in three patients belonging to different haplogroups. This non-synonymous mutation presents only in normal tissues of the patients. The 14288 site itself is not conserved across different species but seems to be quite conserved among human populations; whether this mutation has functional effect is unknown.

**Figure 2:**
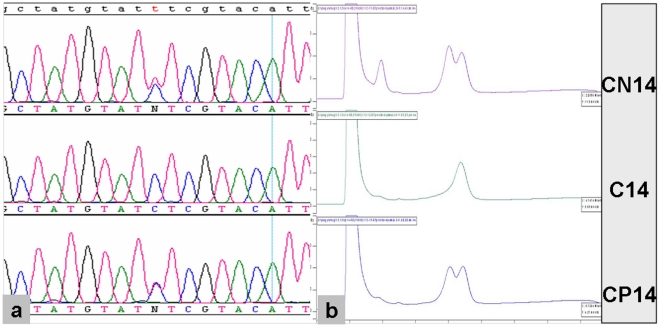
DHPLC assay of the heteroplasmic somatic mutations. Direct sequencing demonstrate that nucleotide site 16093 displays a homoplastic C in cancerous tissue, while in para-cancer normal and distant normal tissue this site is in heteroplasmic status of T/C. DHPLC assay confirmed the sequencing result: in tissue C14 it displays a single peak, whereas in CP14 and CN14 it shows double peak.

In Patient 14, site 16093 in cancerous tissue is homoplastic C, while in para-cancerous normal and distant normal tissue this site shifts to be heteroplasmic as C/T. This pattern was confirmed by DHPLC assay result: in cancerous tissue C14 site 16093 displays a single peak, whereas in para-cancerous normal and distant normal tissues it shows double peak (Figure 2). Of note is that site 16093 itself is a hot spot in the general population and mutation on this site has also been found in normal tissue of cancer patients in other studies [27]. Similar phenomenon also happened to Patient 20, in which only distant normal tissue harbored somatic mutation (viz., A9275G). These mtDNA somatic mutations might be irrelevant to tumorigenesis considering their appearance in the normal tissues.

## Discussion

Hitherto, most of the previous studies on the role of mtDNA in colorectal tumor focused merely on the D-loop or partial segment of the molecule [28], [29], [30], [31], only a few reports have focused on the mutational profile on its whole genome (Polyak et al. 1998). To get more insights into the relationship between mtDNA and colorectal tumor, in the current study we have recruited three tissues (cancerous, para-cancerous and normal tissues) sampled from 20 patients with colorectal tumor; by sequencing the entire mtDNA genome from each tissue and comparing the mutational difference among three tissues from the same patient, our study revealed a relatively low mutational frequency and the heteroplasmic status of all the observed somatic mutations. These observations are quite different from the previous studies, emphasizing the complexity in the etiology of colorectal tumor.

Since most of the patients (12/20) under study do not harbor any mtDNA somatic mutation in their cancerous tissues, one explanation could be that it is the modification in nuclear genome which has contributed to the tumorigenesis in the samples without involving any mtDNA changes. The frequency of mtDNA somatic mutations (6/20; 30%) observed in our current study, which is comparable to our previous results on breast cancer (2/10; 20% [22]), but is significantly lower (p<0.05) than the previous observations, especially those on colorectal tumor. For example, Polyak et al. found that 7/10 (70%) colorectal tumor patients carried one to three mutations [32]. It is possible that the somatic mutation frequency may vary among different ethnic populations or correlate with the cancer stages; testing this possibility would require a systematic design of the study in near future. Alternatively, the much lower incidence of somatic mutations observed in our study is most plausibly attributed to the stringent quality control that was adopted during the data generation and analysis. Indeed, when the potential problems pinpointed in the earlier studies by using a phylogenetic approach [18] were excluded, the frequency of somatic mutations would then decrease considerably.

Intriguingly, all the somatic mutations identified here are in heteroplasmic, which is quite different from the previous observations. Especially, some of the somatic heteroplasmic mutation also present in the normal tissues of the cancer patients. Similarly, Taylor and colleagues also found mtDNA somatic mutations in the normal tissue in colorectal tumor patients, and most of these mutations were heteroplasmic [33]. To evaluate whether the heteroplasmic mtDNA somatic mutations detected in the normal tissues of cancer patients is a normal phenomenon, we investigated the frequency of heteroplasmic mtDNA somatic mutations in the general human populations (embracing a total of 4738 mtDNA genome sequences retrieved from the literature). Our result revealed that the frequency of individuals harboring heteroplasmic somatic mtDNA mutation(s) is less than 4%, suggesting that heteroplasmy in mtDNA from the general population is a rare phenomenon, at least when detected by direct DNA sequencing method [34], [35], [36]. Then we thoroughly reviewed the previous studies on mtDNA mutation in different tumor types and found that heteroplasmic somatic mtDNA mutations were also observed in the normal tissues of cancer patients [9], [22], [27], [33], [37], with a significant higher frequency (38.6%) than in the general population (3.2%; p<0.001) but still lower than that in the cancerous tissue (52.9%; p<0.05) (Table S2). Since each individual is characterized by a mixture of related mitochondrial haplotypes, and widespread heteroplasmy in the mtDNAs of various normal human tissues does have been observed [27], [38], [39], [40]. It is likely that the observed difference in the frequencies of the heteroplasmic mutations between the normal tissue of tumor patients and the general population (commonly adopting their peripheral blood) is attributed to the heteroplasmy between different tissues. Alternatively, the increased heteroplasmic mutation frequency, in both cancerous and matched normal tissues in tumor patients relative to the general population (Table S2), might result from an altered physiological environment (maybe the elevated level of ROS, or some other unknown mutagens). For instance, Goodwin et al. (2008) found that both tumor and matched normal tissues from prostate cancer patients exhibit significantly higher expression of spermine oxidase [41], which has been linked to increased ROS and DNA damage [42], [43], versus tissues from prostate disease-free patients.

In retrospect, previous studies have demonstrated that mtDNA mutation with potential functional effect could contribute to tumor procession [8], [12]. In the present study, the identified somatic mtDNA mutations in coding region all located in evolutionarily highly conserved sites. Therefore, it seems possible that these somatic mutations, albeit all in heteroplasmic, might have played some roles in affecting the function of respiratory chain and thus have some biochemical functional effect to its host cell. However, considering that most of the patients do not harbor somatic mutations in the cancerous tissues, whether the functional effect of these somatic mutations are necessary for the host cell remains unknown. As to the somatic mtDNA mutations present in normal tissues, they should reflect an altered microenvironment for the mtDNA. Whether these mutations, plus the interaction with the altered microenvironment, could increase the susceptibility for a cell to tumorigenesis is unknown.

In total, understanding the relationship between mtDNA somatic mutations and tumorigenesis is a challenging task, because strategies that directly estimating cell fates or tracing the detailed process of both events are still impractical in vivo. By direct comparing mtDNA genome variation in three paired tissues from the same patient, our study could provide an extensive profile of the somatic mtDNA mutation in tumor patient. A low frequency somatic mtDNA mutation was identified in the colorectal patients and all the mutations were heteroplasmic. Meanwhile, some somatic mutations could be present in the normal tissue which indicates that these somatic mutations are not the consequence of tumorigenesis, and it might reflect a changed physiological environment for the mtDNA in tumor patient.

## Materials and Methods

### Clinical samples

A total of 59 tissue samples were collected in this study, including the primary colorectal cancerous tissues (C1–C10), corresponding para-cancerous normal tissues (CP1–CP10) and distant normal tissues (CN1–CN10), which were taken from 20 Chinese patients with colorectal cancer during surgery with informed consent. The cancerous tissue was confirmed by HE-stained slides of the frozen section after surgery, whereas the para-cancerous normal tissue was obtained from the normal tissue surrounding the cancer, and the distant normal tissue is at least 5 cm away from the edge of the cancer tissue. All the patients were clinically diagnosed with stage I (T1–T2, N0, M0) colorectal cancer according to the clinical standard for the category of the cancer [44]. Since the major objective of our study is to pinpoint the subtle difference between cancer and the matched normal tissues from the viewpoint of mitochondrial genome, only the colorectal normal tissues (including the para-cancerous and distant normal tissues) from the same patient were therefore taken into consideration. The para-cancerous normal tissue was not available for Patient 19. The study was performed according to the Declaration of Helsinki.

### DNA amplification and sequencing

Total genomic DNA was respectively extracted from the para-cancerous tissue, primary cancerous tissue and distant normal tissue by using the standard phenol/chloroform method. The entire mtDNA genomes were amplified and sequenced as fully described in our previous studies [19], [20], [21], [22]. Sequencing data were edited and aligned by use of the DNASTAR software (DNAStar Inc., Madison, Wisc.), and mutations were scored relative to the revised Cambridge reference sequence (rCRS) [45]. The phylogenetic status of each mtDNA was determined according to the reconstructed East Asian mtDNA phylogeny [20], [46], and their relationships were illustrated by way of a schematic phylogenetic tree (Figure 1).

### Quality control

To avoid potential problems and errors observed in the published mtDNA disease studies [18], [47], [48], [49], a stringent quality-control procedure [19], [20], [21] was utilized in the course of sample handling and data generation. In addition, to fully avoid artificial recombination triggered by sample mix-up or contamination [18], [50], each tissue sample was amplified and sequenced individually as we have done before [22].

### Determination of heteroplasmy by DHPLC

For the observed heteroplasmic mutation during the direct DNA sequencing, we further confirmed it by denaturing high-performance liquid chromatography (DHPLC) (Figure 2).

## Supporting Information

Table S1 Somatic mtDNA mutations identified in 20 colorectal patients
(DOC)

Table S2 Comparison of the frequency of individuals harboring heteroplasmic mtDNA mutations in normal population and in cancer patients.
(DOC)
