# Supplementary material for: Uncovering the Profile of Somatic mtDNA Mutations in Chinese Colorectal Cancer Patients
Source: PLoS One. 2011 Jun 28;6(6):e21613. doi: 10.1371/journal.pone.0021613 (PMC3125228; doi:10.1371/journal.pone.0021613)
Supplement: Table S1 — Somatic mtDNA mutations identified in 20 colorectal patients (DOC) [file pone.0021613.s001.doc]

Table S1.

| Sample | mutation | Location | Status^$^ | | AA change | haplogroup | Conservation across evolution^#^ | Conservation in human population^*^ |
| --- | --- | --- | --- | --- | --- | --- | --- | --- |
| C1 | 6718 | COI | G->A | He. (A: 60%) | GLY->ASP | B4d | G: 13/13 | G: 3635/3635 |
| CP1 | 6718 | COI | G->A | He. (A: 90%) | GLY->ASP | B4d | G: 13/13 | G: 3635/3635 |
| CP1 | 15332 | Cyt b | C->T | He. (T: 30%). | HIS->TYR | B4d | C: 13/13 | C: 3635/3635 |
| CN1 | 14288 | ND6 | C->A | He. (A: 40%) | GLY->VAL | B4d | C: 5/13 | C: 3635/3635 |
| C3 | 16365 | D-loop | C->T | He. (T: 70%) | Noncoding | F1 | C: 4/13 | C: 3631/3635 |
| CP3 | 16365 | D-loop | C->T | He. (T: 60%) | Noncoding | F1 | C: 4/13 | C: 3631/3635 |
| C4 | 16390 | D-loop | G->A | He. (A: 30%) | Noncoding | A | G: 3/13 | G: 3270/3635 |
| C8 | 15447 | Cyt b | T->C | He. (C: 50%) | LEU->PRO | M7b1 | T: 13/13 | T: 3634/3635 |
| CP8 | 14288 | ND6 | C->A | He. (A: 30%) | GLY->VAL | M7b1 | C: 5/13 | C: 3635/3635 |
| CN8 | 15447 | Cyt b | T->C | He. (C: 60%) | LEU->PRO | M7b1 | T: 13/13 | T: 3634/3635 |
| C12 | 4532 | ND2 | G->A | He. (A: 60%) | ALA->ALA | M8a2 | G: 2/13 | G: 3633/3635 |
| CP14 | 16093 | D-loop | T->C | He. (C: 40%) | Noncoding | F1 | T: 4/13 | T: 3286/3635 |
| CN14 | 14288 | ND6 | C->A | He. (A: 30%) | GLY->VAL | F1 | C: 5/13 | C: 3635/3635 |
| CN14 | 16093 | D-loop | T->C | He. (C: 40%) | Noncoding | F1 | T: 4/13 | T: 3286/3635 |
| C16 | 215 | D-loop | A->G | He. (G: 50%) | Noncoding | F2 | A: 5/13 | A: 3604/3635 |
| C16 | 15276 | Cyt b | G->A | He. (A: 40%) | ARG->GLU | F2 | G: 13/13 | G: 3635/3635 |
| C16 | 16158 | D-loop | A->G | He. (G: 50%) | Noncoding | F2 | A: 6/13 | A: 3613/3635 |
| CN20 | 9275 | COIII | A->G | He. (G: 40%) | SER->SER | M13a | A: 9/13 | A: 3632/3635 |

Note: He.: heteroplasmic

$: the percentage of one allele on one site was estimate according to the highness of the peak of this allele in sequencing chromatogram, also confirmed in DHPLC assessment.

#: the conservation was evaluated in an alignment of the mitochondrial genomes from representative 13 vertebrate including: *Homo sapiens*; *Gorilla gorilla; Pan troglodytes; Macaca mulatta; Canis familiaris; Bos Taurus; Balaenoptera musculus; Rattus norvegicus; Mus musculus; Gallus gallus; Iguana iguana; Xenopus laevis; Danio rerio.*

*: We evaluate the conservation of each nucleotide position of these somatic mutations in 3635 complete mitochondrial genome sequences of human general population reported previously.
