# Supplementary material for: Uncovering the Profile of Somatic mtDNA Mutations in Chinese Colorectal Cancer Patients
Source: PLoS One. 2011 Jun 28;6(6):e21613. doi: 10.1371/journal.pone.0021613 (PMC3125228; doi:10.1371/journal.pone.0021613)
Supplement: Table S2 — Comparison of the frequency of individuals harboring heteroplasmic mtDNA mutations in normal population and in cancer patients. (DOC) [file pone.0021613.s002.doc]

Table S2.

| Type | Frequency ( normal population) | Frequency(normal tissue of cancer patients) | frequency (cancer tissue of cancer patients) | Reference |
| --- | --- | --- | --- | --- |
| Pedigree | **2.88%** (6/208) | —— | —— | (Santos et al. 2008) |
| Pedigree | **2.37%** (10/422) | —— | —— | (Santos et al. 2005) |
| Twins plus unrelated individuals | **1.48%** (7/473) | —— | —— | (Bendall et al. 1996) |
| unrelated population | **3.16%** (115/3635) | —— | —— | data collected in present study**^#^** |
| Breast cancer patients | —— | **10%** (1/10) | **20%** (2/10) | (Wang et al. 2007) |
| Colorectal cancer patients | —— | **25%** (5/20) | **30%** (6/20） | Present study |
| Colorectal cancer patients | —— | **40%** (4/10) | **70%** (7/10) | (He et al. 2010)**^*^** |
| Prostate cancer patients | —— | **79.2%**(19/24) | **91.7%** (22/24) | (Parr et al. 2006) |

Note:

#: we collected 3635 complete mitochondrial sequences of normal human population from the previous published studies, and the accession number of these sequences in GenBank can be acquired on request to the authors.

*: Digital sequencing is applied in this study and a lot of heteroplasmic mutations were found in patients. It’s suggested that heteroplasmic point mutations with a 10%-20% or lower percentage for the minor allele were likely to be missed during direct sequencing (Bendall et al. 1996), thus here we take >25% for the minor allele as the criteria which can be detected by direct sequencing in security.
